# The importance of *Haemophilus influenzae* in community-acquired pneumonia: an emerging pathogen in the elderly regardless of comorbidities compared to *Streptococcus pneumoniae*

**DOI:** 10.1186/s41479-024-00136-w

**Published:** 2024-08-25

**Authors:** Linda Yamba Yamba, Karin Hansen, Lisa Wasserstrom, Yu-Ching Su, Jonas Ahl, Kristian Riesbeck

**Affiliations:** 1 Clinical Microbiology, Department of Translational Medicine, Faculty of Medicine, Lund University, Jan Waldenströms Gata 59, 205 02 Malmö, Sweden; 2 Infectious Diseases, Department of Translational Medicine, Faculty of Medicine, Lund University, Malmö, Sweden; 3 Clinical Microbiology, Laboratory Medicine Skåne, Infection Control and Prevention, Lund, Sweden

**Keywords:** CAP, Community acquired pneumonia, *Haemophilus influenzae*, Pneumonia, *Streptococcus pneumoniae*

## Abstract

**Background:**

*Haemophilus influenzae* community-acquired pneumonia (CAP) is common, and it is equally common to *Streptococcus pneumoniae* in some settings. The purpose of this study was to provide additional data on patients affected by *H. influenzae* CAP and their outcomes.

**Methods:**

*Streptococcus pneumoniae*-caused CAP (111 cases) was compared to CAP with *H. influenzae* (53 cases). Patients were adults (≥ 18 years) from the prospective study “Etiology of community acquired pneumonia in Sweden” (ECAPS), which was established during the years 2016–2018.

**Results:**

Cases with *H. influenzae* CAP were significantly older compared to *S. pneumoniae* CAP (median 77 vs 70 years, *p* = 0.037) albeit similar comorbidities. *Haemophilus influenzae* was generally absent in the bloodstream compared to *S. pneumoniae* (18% vs 2%, *p* = 0.01) but clinical presentations were comparable. Only a minority of patients, 34% with *H. influenzae* and 41% with *S. pneumoniae* CAP had underlying lung disease.

**Conclusion:**

In the light of childhood immunization campaigns against *S. pneumoniae* and the increasing numbers of pneumococcal vaccinations among the elderly, coupled with an aging population, the incidence of CAP caused by *H. influenzae* may increase. Further research is needed to understand the impact of *H. influenzae* CAP and to a development of a vaccine against this emerging microbe.

**Supplementary Information:**

The online version contains supplementary material available at 10.1186/s41479-024-00136-w.

## Background

*Streptococcus pneumoniae* has been considered the primary bacterial cause of community-acquired pneumonia (CAP) [1]. Recent studies using molecular methods and comprehensive testing have, however, further highlighted the importance of *Haemophilus influenzae* in CAP, indicating equal rates of *H. influenzae* (24–41%) involvement compared to *S. pneumoniae* (21–35%) [2, 3]. During the twentieth century, *H. influenzae* type b (Hib) was a common cause of invasive disease. Following the introduction of Hib vaccination in the 1990s, the incidence of serotype b has decreased, and now invasive as well as respiratory disease is primarily caused by non-typeable Hi and in rare cases other serotypes than b [4, 5].

To provide additional data on CAP caused by *H. influenzae* after the implementation of conjugated pneumococcal vaccine (PCV) in 2009, a comparison was made between CAP cases in our setting caused by *S. pneumoniae,* the most common bacterial species 28%, and *H. influenzae* 16%, the second most common species [6, 7]. In 2009 PCV7 was introduced but quickly changed to PCV10 in 2010. During the following years mainly PCV10 (2010–2014, 2018–2023) and PCV13 (2014–2018) has been used in the children’s immunization programme in Skane county. Our aim was to provide further insight into the affected patient populations and observed outcomes that are associated with *H. influenzae* CAP.

## Methods

### Patients

This is a sub-analysis of the “Etiology of community-acquired pneumonia in Sweden” (ECAPS) cohort, which was a prospective case control study including 567 patients and 500 controls between September 2016-September 2018 at Skåne University Hospital [6, 7]. Eligible patients in ECAPS were ≥ 18 years of age, had 2 out of 10 predefined symptoms of pneumonia, radiographic finding indicating pneumonia, was included within 48 h, and, finally, provided a urine sample. In contrast, exclusion criteria were hospitalization or pneumococcal vaccination < 30 days prior to admission and previous enrolment. In the current study, adult patients (*n* = 518) from the ECAPS cohort based on detection of *S. pneumoniae* or *H. influenzae*, either with or without concurrent viral detection (Supplementary Fig. 1) [6, 7]. Exclusion criteria included the detection of multiple bacterial pathogens in a patient or lack of diagnostic testing using real-time PCR per protocol. Additional information about microbiological testing is outlined in the Supplementary Data. These criteria resulted in the inclusion of 164 patients (22–96 years of age). Severity assessment was conducted using the Pneumonia Severity Index (PSI), with class IV-V indicating a moderate to high risk of mortality. Mortality was measured using case fatality rates (CFR) at 30 or 90 days. The radiological findings were classified based on the report made by the radiologist on duty.

### Statistical analysis

Statistical analysis was performed using RStudio 4.3.0. For the comparison of proportions, Pearson’s chi-square-/Fisher’s exact test was used. For group comparisons of continuous data, Kruskal Wallis and Mann Whitney test or ANOVA and *t*-tests were performed. A *p*-value below 0.05 was regarded as statistically significant, and we made Bonferroni corrections if multiple comparisons were performed.

## Results

A total of 164 patients were included, with the majority having CAP caused by *S. pneumoniae* (*n* = 111) and the remaining cases caused by *H. influenzae* (*n* = 53). *Haemophilus influenzae* CAP patients had a significantly higher age (median 77 years) compared to *S. pneumoniae* CAP patients (70 years). Male sex was more prevalent among *S. pneumoniae* CAP, while female sex was more common among *H. influenzae* cases. Most comorbidities, including COPD and asthma, were equally common among both patient groups (Table 1). No significant differences were observed regarding symptoms of pneumonia at admission (Table 2).

**Table 1:** Patient characteristics and outcomes for different CAP etiologies

	**All**	***S. pneumoniae***	***H. influenzae***	
	*n* = 164	*n* = 111	*n* = 53	***p***
**Age (years)**, median [IQR]	73 (62,83)	70 (59,83)	77 (68,84)	**0.037**
**Female sex**, *n* (%)	77 (47)	48 (43)	29 (55)	0.22
**Body mass index**, mean (SD)	25.4 (5.7)	25.0 (5.5)	26.3 (6.1)	0.18
**Smoker**				0.07
No, *n* (%)	50 (30)	34 (31)	16 (30)	
Yes, *n* (%)	33 (20)	28 (25)	5 (9.4)	
Previous smoker, *n* (%)	81 (49)	49 (44)	32 (60)	
**Pneumococcal vaccination** *n*/*N *^*a*^ (%)	19/157 (12)	15/105 (14)	4/52 (7.7)	0.3
**Influenza vaccination**, *n*/*N* ^b^ (%)	67/160 (42)	43/108 (40)	24/52 (46)	0.56
**Chronic pulmonary disease**, *n* (%)	63 (39)	45 (41)	18 (34)	0.41
**COPD**, *n*/*N* (%)	55/163 (34)	38/110 (35)	17 (32)	0.89
**Asthma**, *n* (%)	17 (10)	11 (10)	6 (11)	> 0.99
**Congestive heart failure**, *n* (%)	22 (13)	16 (14)	6 (11)	0.77
**Coronary artery disease**, *n* (%)	39 (24)	28 (25)	11 (21)	0.67
**Dementia**, *n* (%)	5 (3.0)	1 (0.9)	4 (7.5)	**0.04**
**Autoimmune disease**, *n* (%)	12 (7.3)	8 (7.2)	4 (7.5)	> 0.99
**Diabetes**, *n* (%)	25 (15)	15 (14)	10 (19)	0.5
**Liver disease**, *n* (%)	6 (3.7)	4 (3.6)	2 (3.8)	> 0.99
**Immunosuppression therapy**, *n*/*N* (%)	22/163 (13)	13 (12)	9/52 (17)	0.47
**Chronic kidney disease**, *n* (%)	15 (9.1)	8 (7.2)	7 (13)	0.25
**Immunodeficiency** ^c^, *n* (%)	1 (0.6)	1 (0.9)	0 (0)	-
**Cancer—solid tumour**, *n* (%)	38 (23)	28 (25)	10 (19)	0.48
**Cancer—hematologic**, *n* (%)	6 (3.7)	5 (4.5)	1 (1.9)	0.67
**Organ transplantation**, *n* (%)	1 (0.6)	1 (0.9)	0 (0)	> 0.99
**Alcohol abuse**, *n* (%)	8 (4.9)	7 (6.3)	1 (1.9)	0.28
**Illicit drug use**, *n* (%)	4 (2.4)	2 (1.8)	2 (3.8)	0.59
**Length of stay**, median [IQR]	5.5 (4.0,8.0)	5.0 (4.0,8.0)	6.0 (4.0,9.0)	0.17
**CRB-65** 3–4, *n* (%)	1 (0.6)	0 (0)	1 (1.9)	-
**PSI** grade IV-V, *n* (%)	84 (51)	57 (51)	27 (51)	> 0.99
**PSI score,** median [IQR]	93 (69,114)	93 (68,114)	92 (74,109)	0.5
**Case fatality rate 30 days**, *n* (%)	6 (3.7)	3 (2.7)	3 (5.7)	0.39
**Case fatality rate 90 days**, *n* (%)	8 (4.9)	4 (3.6)	4 (7.5)	0.27
**Intensive care admission**, *n* (%)	3 (1.8)	1 (0.9)	2 (3.8)	0.24

Significant differences between the two groups (*H. influenzae* and *S. pneumoniae* CAP) are indicated with bold* p*-values

*Abbreviations*: *COPD *Chronic obstructive pulmonary disease, *CRB-*65 Confusion-respiratory-breathing-65 score, *IQR* Interquartile range, *PSI *pneumonia severity index

^a^n/N: number of total patients sampled. Not all individuals were completely sampled resulting in some missing data

^b^Vaccination against influenza within the last year prior to CAP

^c^Known immunodeficiency including HIV or AIDS

**Table 2:** Symptoms, clinical findings and viral codetection in CAP caused by *Streptococcus pneumoniae* or *Haemophilus influenzae*

	**Total samples**	***S. pneumoniae***	***H. influenzae***	
	*n* = 164	*n* = 111	*n* = 53	***p***
**Symptoms and Clinical findings**				
Fever, *n* (%)	147 (90)	100 (90)	47 (89)	> 0.99
Chills or rigor, *n* / *N* ^a^ (%)	105/163 (64)	76 (68)	29/52 (56)	0.16
Pleuritic chest pain, *n* (%)	68 (41)	50 (45)	18 (34)	0.24
Cough, *n* (%)	146 (89)	96 (86)	50 (94)	0.22
Sputum, *n* / *N* (%)	97/163 (60)	65 (59)	32/52 (62)	0.85
Dyspnoea, *n* (%)	127 (77)	86 (77)	41 (77)	> 0.99
Tachypnoea, *n* (%)	100 (61)	71 (64)	29 (55)	0.34
Malaise, *n* (%)	144 (88)	96 (86)	48 (91)	0.62
Auscultatory finding, *n* (%)	123 (75)	85 (77)	38 (72)	0.7
**Radiological finding**, *n* (%)				
Lobar infiltrate unilateral	81 (49)	65 (59)	16 (30)	**0.005** **-**
Lobar infiltrate bilateral	31 (19)	16 (14)	15 (28)	0.21
Interstitial infiltrate unilateral	15 (9.1)	13 (12)	2 (3.8)	0.29
Interstitial infiltrate bilateral	14 (8.5)	6 (5.4)	8 (15)	0.21
Peribronchial infiltrate	1 (0.6)	1 (0.9)	0 (0)	> 0.99
Other finding indicating pneumonia	22 (13)	10 (9.0)	12 (23)	0.13
**Viral finding**, *n* (%)	54 (33)	41 (37)	13 (25)	0.16
Entero-/ rhinovirus	28 (17)	19 (17)	9 (17)	> 0.99
Coronavirus	4 (2.4)	4 (3.6)	0 (0)	0.31
Influenza A	7 (4.3)	5 (4.5)	2 (3.8)	> 0.99
Influenza B	4 (2.4)	3 (2.7)	1 (1.9)	> 0.99
Human metapneumovirus	6 (3.7)	5 (4.5)	1 (1.9)	0.67
RSV A/B	6 (3.7)	6 (5.4)	0 (0)	0.18
Parainfluensavirus 1 to 3	1 (0.6)	1 (0.9)	0 (0)	> 0.99
Adenovirus	0 (0)	0 (0)	0 (0)	- -
Parechovirus	0 (0)	0 (0)	0 (0)	-

^a^*n*/*N* if data is missing

Length of stay, CRB-65, PSI grade IV-V and PSI mean score were comparable between the groups (Table 1). These findings remained consistent even when stratifying based on viral co-detection (Supplementary Table 1). *Haemophilus influenzae* CAP case fatality was not significantly higher than *S. pneumoniae* at 30 (5.7% vs 2.7%, *p* = 0.39) or 90 days (7.5% vs 3.6%, *p* = 0.27). *Haemophilus influenzae* CAP cases had a higher, but not significant, incidence of admission to the intensive care unit compared to *S. pneumoniae* CAP cases (3.8% vs 0.9%). A positive blood culture was significantly more common in *S. pneumoniae* CAP patients compared to *H. influenzae* CAP (18% vs 2%, *p* = 0.01). The majority of *H. influenzae* cases were detected by nasopharyngeal PCR (89%) or culture (81%). This was in contrast to *S. pneumoniae;* most of the cases were detected by UAD (66%) followed by nasopharyngeal PCR (60%) (Table 3). A minority of the patients in our cohort had a viral co-infection, accounting for 37% of *S. pneumoniae* cases and 25% of *H. influenzae* cases. The most detected viruses were Entero-/ rhinoviruses 17% in both patient groups followed by influenza virus. Among patients with pneumococcal pneumonia, RSV A/B was found in 5.4% of cases compared to none among patients with *H. influenzae* (Table 2).

**Table 3:** Microbiological diagnostic findings of CAP patients. Significant differences between the two groups (*H. influenzae* and *S. pneumoniae* CAP) are indicated with bold* p*-values

	**All**	***S. pneumoniae***	***H. influenzae***	
	*n* = 164	*n* = 111	*n* = 53	***p***
**Bacterial diagnostic findings**^**a**^				
Positive blood culture, *n*/*N* (%) ^b^	20/156 (13)	19/105 (18)	1/51 (2)	**0.01**
Positive nasopharyngeal culture, *n*/*N* (%)	46/128 (36)	12/86 (14)	34/42 (81)	** < 0.001**
Positive *H. influenzae* NP PCR ^c^, *n*/*N* (%)	-	-	47/53 (89)	-
Positive *S. pneumoniae* NP PCR, *n* (%)	-	67 (60)	-	-
Positive UAD, *n* (%)	-	73 (66)	-	-
Positive BINAX *S. pneumoniae*®*, n* (%)	-	52 (47)	-	-

^a^In addition, two lower respiratory tract samples were also culture positive; one for *S. pneumoniae* and one for *H. influenzae*

^b^n/N: number of total patients sampled. Not all individuals were completely sampled resulting in some missing data

^c^*Abbreviations*: *NP *nasopharyngeal, *PCR *polymerase chain reaction, *UAD* pneumococcal serotype specific urine antigen detection assay

## Discussion

The presence of underlying lung disease was equally common in patients with CAP caused by *S. pneumoniae* and *H. influenzae*, observed in 39% of the patients. This finding was surprising as previous studies on lower respiratory tract infections by *H. influenzae* have indicated that underlying lung diseases, such as COPD are the main risk factors, with a prevalence typically over 50% [8, 9]. Our earlier study on bacteremia and lower respiratory tract infection caused by *H. influenzae* in Skane county (year 1997–2016) also revealed a lower prevalence of COPD at 25% [10]. In addition, the 30-day mortality when excluding bacteremia cases, was comparable to the present study at 7%. Of importance is that even if *H. influenzae* is common and important to acknowledge for patients with respiratory disease, its ability to infect and cause severe disease in other individuals is not negligible. Patients with *H. influenzae* CAP had a comparable case fatality rate compared to pneumococcal pneumonia patients in our study. Furthermore, stratification based on concurrent viral infection did not show increased morbidity for either pathogen, contrary to observations by Shoar et al*.* [9]. Benzylpenicillin is recommended as empirical treatment for mild to moderate CAP in Sweden, a practice that can be questioned due to the relatively common occurrence of Benzylpenicillin (Penicillin G) resistance in *H. influenzae*. A recent study from us did not, however, show any increased mortality with Benzylpenicillin compared to other beta-lactam antibiotics [10]. One important fact to acknowledge is also the increased virulence of certain non-typeable *H. influenzae* strains that may result in severe infections [11]. Of the few individuals with *H. influenzae* CAP (*n* = 4) that died during the following 3 months, half were over 80 years of age, but none had any known immunodeficiencies or immunosuppressive therapy.

Patients with *H. influenzae* CAP were significantly older than those with *S. pneumoniae* CAP. Older patients are at increased risk of CAP, severe outcomes, and negative effects on the quality of life after admission [12, 13]. Life expectancy is increasing, and projections say the European Union will have over 64 million habitants over the age of 80 in 2100 [14]. When a larger proportion of society is at risk of severe disease, *H. influenzae* CAP may become more common in a clinical setting without preventive measures.

It is debated if pneumococcal conjugated vaccines have increased the carriage of non-typable *H. influenzae* in children [15]. In contrast, no change in *H. influenzae* carriage among adults has been observed following adult pneumococcal vaccination with the 13-Valent Pneumococcal Conjugate Vaccine [16]. One study has also implicated an increased risk of *H. influenzae* CAP associated with pneumococcal vaccination [8]. *Haemophilus influenzae* is frequently carried by adults in the nasopharynx. In the ECAPS cohort the included asymptomatic control group (*n* = 241, median age 64) had a nasopharyngeal carriage rate, tested with real-time PCR, of 6% compared to 14% (*p* = 0.001) among CAP patients (*n* = 518, median age 73) [7]. Further research is necessary to comprehensively assess the potential impact of widespread pneumococcal vaccination on the incidence of lower respiratory tract diseases caused by *H. influenzae* in various settings.

Our study has limitations. Firstly, most of our findings regarding *H. influenzae* CAP is based on nasopharyngeal culture or PCR, whereas other studies rely on lower respiratory tract samples. In Sweden, treatment guidelines recommend the use of nasopharyngeal samples for CAP diagnosis, as sputum sampling and culture in many cases is cumbersome [17]. *Haemophilus influenzae* is, however, more likely to be detected in the nasopharyngeal tract of symptomatic adults than in healthy adults, where the bacterium is found in smaller numbers [6, 18]. Secondly, we did not exclude that encapsulated *H. influenzae* occurs amongst the isolates. The vast majority of *H. influenzae* in the Swedish population is non-typeable bacteria, however, since a nationwide vaccine against encapsulated *H. influenzae* type b (Hib) was included in the child immunization programme in the early 1990s. Data on Hib in respiratory tract infections in Sweden is not available, but the incidence regarding invasive disease is very low; 0.8–2.5 cases/ 100,000 inhabitants (2014–2023) [19]. Despite other serotypes including serotype f (Hif) may occur they are very rare in Sweden. Therefore, we can postulate that patients with diagnosed *H. influenzae* in most cases suffer from non-typeable, that is unencapsulated *H. influenzae* [4]. Thirdly, there is a possibility that ICU-admitted patients were not extensively enrolled, as initial consent may not have been possible, potentially resulting in missing cases with severe presentations upon hospital admission [6]. In addition, the patient number in our current setting was relatively low, limiting the power of statistical analysis which highlights the need for larger studies on *H. influenzae* CAP.

Patients with *H. influenzae* CAP exhibit similar comorbidities to those susceptible to *S. pneumoniae* CAP, and hospital admission is not solely associated with underlying respiratory diseases. *Haemophilus influenzae* CAP cases were significantly older but outcomes comparable. As the population continues to age, the significance of *H. influenzae* CAP may rise, especially if *S. pneumoniae* CAP rates decline due to expanded vaccination among adult risk groups. We suggest that, amidst the emphasis on research to prevent pneumococcal disease, the potential and existing burden of *H. influenzae* should not be overlooked, and there should be room for a vaccine against *H. influenzae* amongst the aging population.

### Supplementary Information

Supplementary Material 1.

## Data Availability

No datasets were generated or analysed during the current study.

## Abbreviations

CAP: Community acquired pneumonia
COPD: Chronic obstructive pulmonary disease
CRB-65: Confusion-respiratory-breathing-65 score
ECAPS: Etiology of community acquired pneumonia in Sweden
Hib: *Haemophilus influenzae* Type b
PCR: Polymerase chain reaction
PCV: Pneumococcal conjugated vaccine
PSI: Pneumonia severity index
