# Supplementary material for: The importance of Haemophilus influenzae in community-acquired pneumonia: an emerging pathogen in the elderly regardless of comorbidities compared to Streptococcus pneumoniae
Source: Pneumonia (Nathan). 2024 Aug 25;16:15. doi: 10.1186/s41479-024-00136-w (PMC11344911; doi:10.1186/s41479-024-00136-w)
Supplement: Supplementary file 1 — Supplementary Material 1. [file 41479_2024_136_MOESM1_ESM.docx]

**Supplementary data**

**The importance of *Haemophilus influenzae* in community-acquired pneumonia: an emerging pathogen in the elderly regardless of comorbidities compared to *Streptococcus pneumoniae***

Linda Yamba Yamba^1^, Karin Hansen^1,2^, Lisa Wasserstrom^3^, Yu-Ching Su^1^, Jonas Ahl^1,2^ and Kristian Riesbeck^1,3^

*^1^Clinical Microbiology, Department of Translational Medicine, Faculty of Medicine, Lund University, Malmö, Sweden*

*^2^Infectious Diseases, Department of Translational Medicine, Faculty of Medicine, Lund University, Malmö, Sweden*

*^3^Clinical Microbiology, Infection Control and Prevention, Laboratory Medicine, Lund, Sweden*

**Corresponding author**

Professor Kristian Riesbeck. e-mail: [kristian.riesbeck@med.lu.se](mailto:kristian.riesbeck@med.lu.se), Phone: +46 730 377 433, Fax: N/A, Jan Waldenströms gata 59, SE-205 02 Malmö, Sweden

**Microbiological testing**

Results from cultures, urine antigens and PCR analysis performed on clinical indication were collected. Cultures performed were mainly collected from blood (156/164 tested) and the nasopharynx (128/164 tested). A smaller number of individuals 10/164 also had one or several lower respiratory tract cultures performed. Patients had to provide a urine sample for inclusion, and this was to analyse the urine at Pfizer’s Vaccines Research and Development Laboratory (Pearl River, NY). Both a pneumococcal urinary antigen, BinaxNOW *S pneumoniae*^®^ (Abbott Diagnostics, Scarborough, ME), and two serotype-specific urinary antigen tests were used as described by Hansen *et al* (1-4).

Upon inclusion to the study an additional nasopharyngeal flocked swab was collected, and a Real-time PCR conducted per protocol to detect 6 bacterial and 14 viral respiratory pathogens listed in Supplemental Figure 1. Several different PCR schemes were used.

Realtime-PCR amplifications for viral agents were performed on an ABI 7500 real-time PCR system (Applied Biosystems, Waltham, MA) in 5 multiplex reactions, adopted from Østby *et al*., and Ek *et al*., containing viruses presented in Supplementary Figure 1 (5-6).

Real-time PCR amplification for bacterial detection were performed using a Bio-Rad CFX96 (Bio-Rad, Hercules, CA) and SensiFAST Probe No-ROX Kit (Bioline, Meridian Bioscience, Cincinnati, OH).

The PCR for *S. pneumoniae* was an in-house method developed at the Clinical Microbiology (Laboratory Medicine Skåne). The protocol includes a forward primer LytA-1-F 5’- GCTGGGTCAAGTACAAGGACACT-3, a reverse primer LytA-1-R 5’- GTCCGCTGACTGGATAAAGGCA-3’ and the probe Lyt-P 5’- FAM- ACTTAGACGCTAAAGAAGGCGCCATGGTATC-BHQ-1-3’.

The PCR for *H. influenzae, M. pneumoniae*, *C. pneumoniae, B. pertussis* and *B. parapertussis* was performed as previously described (7-10). Moreover, *H. influenzae* was additionally verfied by PCR targeting a species-specific outer membrane protein (manuscript in preparation).

**Supplementary Figure 1.** Inclusion of patients from the ECAPS cohort.


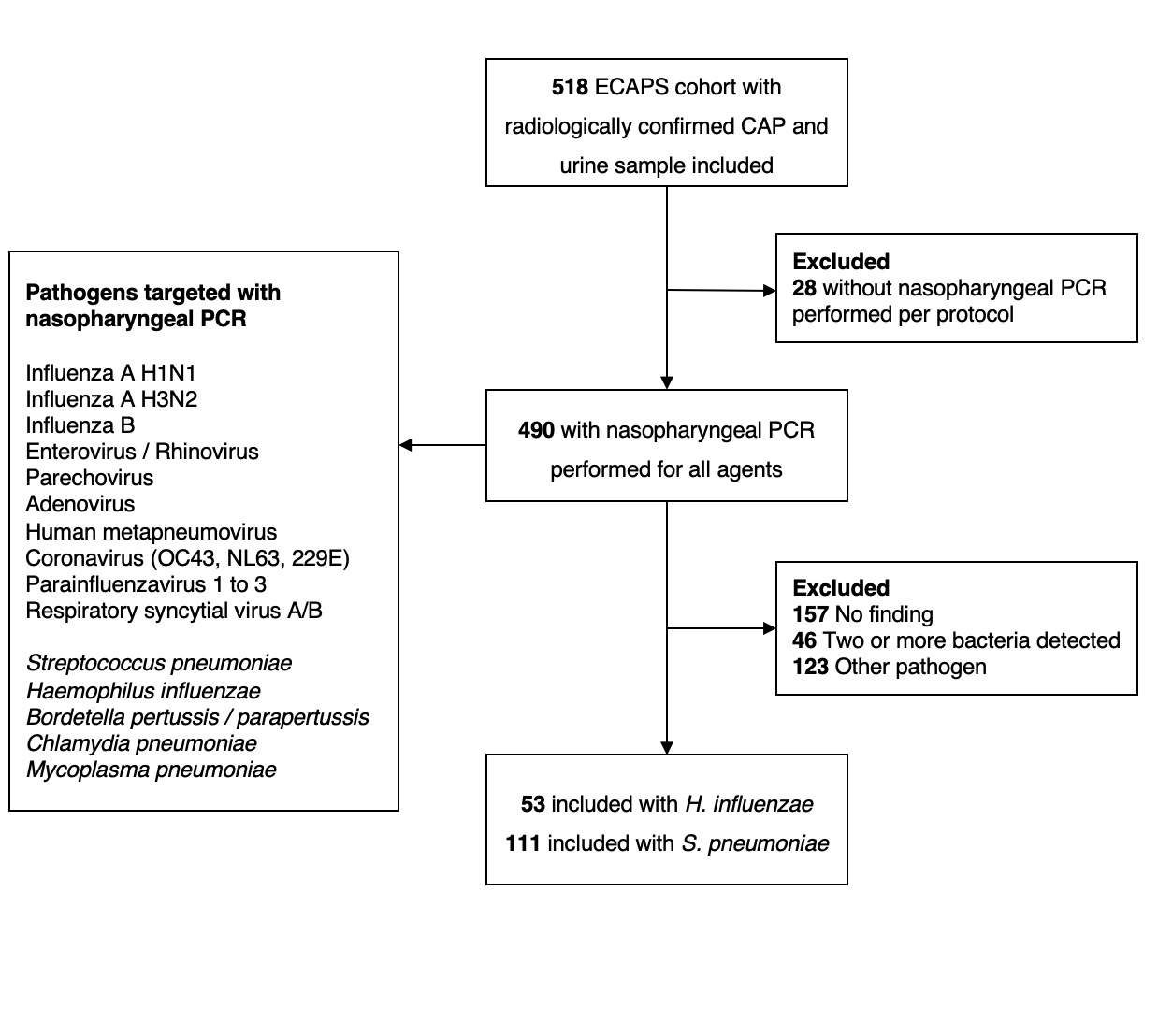


**Supplementary Table 1.** Outcome and severity depending on viral co-infection addition in CAP caused by *Streptococcus pneumoniae* and *Haemophilus influenzae*.

|  | **All** | ***S. pneumoniae*** | | ***H. influenzae*** | |  |
| --- | --- | --- | --- | --- | --- | --- |
|  |  | Single | + Virus | Single | + Virus | ***p*** |
|  | *n* = 164 | *n* = 70 | *n* = 41 | *n* = 40 | *n* = 13 |  |
| **Length of stay** Median [IQR] ^a^ | 5.5 [4–8] | 5 [4–7] | 5.0 [4–9] | 6 [5–8] | 6 [4–9] | 0.52 |
| **CRB-65** 3-4, *n* (%) | 1 (0.6) | 0 (0) | 0 (0) | 1 (2.5) | 0 (0) | - |
| **PSI** grade IV-V, *n* (%) | 84 (51) | 34 (49) | 23 (56) | 20 (50) | 7 (54) | 0.89 |
| **PSI score**, Median [IQR] | 93 [69–114] | 88 [64–114] | 94 [78–114] | 91 [74–108] | 108 [75–125] | 0.58 |
| **Case fatality rate 30 days** | 6 (3.7) | 2 (2.9) | 1 (2.4) | 3 (7.5) | 0 (0) | 0.56 |
| **Case fatality rate 90 days** | 8 (4.9) | 2 (2.9) | 2 (4.9) | 4 (10) | 0 (0) | 0.39 |

^a^ Abbreviations: **PSI** - Pneumonia severity index – grade IV-V indicates a prediction of higher mortality; **CRB-65 –** grade 3-4 indicates a prediction of higher mortality; **IQR** – Interquartile range.

**References**

1. Hansen K, Rünow E, Torisson G, Theilacker C, Palmborg A, Pan K, et al. Radiographically confirmed community-acquired pneumonia in hospitalized adults due to pneumococcal vaccine serotypes in Sweden, 2016-2018-The ECAPS study. Frontiers in Public Health. 2023;11:1086648.
2. Hansen K, Yamba Yamba L, Wasserstrom L, Rünow E, Göransson T, Nilsson A, et al. Exploring the microbial landscape: uncovering the pathogens associated with community-acquired pneumonia in hospitalized patients. Frontiers in Public Health. 2023;11.
3. Pride MW, Huijts SM, Wu K, Souza V, Passador S, Tinder C, et al. Validation of an immunodiagnostic assay for detection of 13 Streptococcus pneumoniae serotype-specific polysaccharides in human urine. Clin Vaccine Immunol. 2012;19(8):1131-41.
4. Kalina WV, Souza V, Wu K, Giardina P, McKeen A, Jiang Q, et al. Qualification and Clinical Validation of an Immunodiagnostic Assay for Detecting 11 Additional Streptococcus pneumoniae Serotype-specific Polysaccharides in Human Urine. Clin Infect Dis. 2020;71(9):e430-e8.
5. Ek P, Böttiger B, Dahlman D, Hansen KB, Nyman M, Nilsson AC. A combination of naso- and oropharyngeal swabs improves the diagnostic yield of respiratory viruses in adult emergency department patients. Infect Dis (Lond). 2019;51(4):241-8.
6. Østby AC, Gubbels S, Baake G, Nielsen LP, Riedel C, Arpi M. Respiratory virology and microbiology in intensive care units: a prospective cohort study. Apmis. 2013;121(11):1097-108.
7. Smith-Vaughan H, Byun R, Nadkarni M, Jacques NA, Hunter N, Halpin S, et al. Measuring nasal bacterial load and its association with otitis media. BMC Ear Nose Throat Disord. 2006;6:10.
8. Hardegger D, Nadal D, Bossart W, Altwegg M, Dutly F. Rapid detection of Mycoplasma pneumoniae in clinical samples by real-time PCR. J Microbiol Methods. 2000;41(1):45-51.
9. Welti M, Jaton K, Altwegg M, Sahli R, Wenger A, Bille J. Development of a multiplex real-time quantitative PCR assay to detect Chlamydia pneumoniae, Legionella pneumophila and Mycoplasma pneumoniae in respiratory tract secretions. Diagn Microbiol Infect Dis. 2003;45(2):85-95.
10. Roorda L, Buitenwerf J, Ossewaarde JM, van der Zee A. A real-time PCR assay with improved specificity for detection and discrimination of all clinically relevant Bordetella species by the presence and distribution of three Insertion Sequence elements. BMC Research Notes. 2011;4(1):11.
